# Integrated Environmental Cadmium Exposure, Plaque Calcium Content, and Lifestyle Factors Improve Prediction of Carotid Plaque Echogenicity: An Exposome-Oriented Approach

**DOI:** 10.3390/toxics14080707

**Published:** 2026-08-11

**Authors:** Adam Płoński, Aleksandra Jurczuk, Adam Filip Płoński, Małgorzata Gałażyn-Sidorczuk, Aleksandra Konopka, Karol Struniawski, Marcin Gabriel, Adam Siebieszuk, Maria Jurczuk

**Affiliations:** 1 Department of Vascular Surgery and Transplantation, Medical University of Bialystok, 15-276 Bialystok, Poland; adam.plonski@sd.umb.edu.pl; 2 Department of Clinical Immunology, Medical University of Bialystok, 15-274 Bialystok, Poland; aleksandra.jurczuk@sd.umb.edu.pl; 3 Department of Toxicology, Medical University of Bialystok, 15-222 Bialystok, Poland; malgorzata.galazyn-sidorczuk@umb.edu.pl (M.G.-S.); maria.jurczuk@umb.edu.pl (M.J.); 4 Institute of Information Technology, Warsaw University of Life Sciences, 02-776 Warsaw, Poland; aleksandra_konopka@sggw.edu.pl (A.K.); karol_struniawski@sggw.edu.pl (K.S.); 5 Department of Vascular and Intravascular Surgery, Angiology and Phlebology, Poznan University of Medical Science, 61-701 Poznan, Poland; mgabriel@pro.onet.pl; 6 Department of Physiology, Medical University of Bialystok, 15-222 Bialystok, Poland; adam.siebieszuk@gmail.com

**Keywords:** cadmium, calcium, carotid plaque, atherosclerosis, Gray-Scale Median (GSM), plaque echogenicity, diet, smoking, logistic regression

## Abstract

Cadmium exposure has been implicated in the development of atherosclerosis, yet its relationship with carotid plaque composition and echogenicity remains poorly understood. A better understanding of the environmental and biological factors influencing plaque characteristics may facilitate more accurate stratification of patients at high risk of ischemic stroke. The aim of this study was to evaluate the relationships between environmental cadmium exposure, plaque calcium content, lifestyle-related factors, and carotid plaque echogenicity, and to assess their combined value for predicting a highly echogenic carotid plaque phenotype. This cross-sectional study included 80 patients with advanced carotid atherosclerosis undergoing carotid endarterectomy. Cadmium concentrations were simultaneously determined in blood, urine, and surgically excised carotid plaques, whereas calcium concentrations were measured in plaque tissue, serum, and urine using atomic absorption spectrometry. Carotid plaque echogenicity was assessed preoperatively using ultrasound-derived Gray-Scale Median (GSM), and multivariable logistic regression models were developed to predict a highly echogenic, stable plaque phenotype. Current smokers exhibited significantly higher blood and urinary cadmium concentrations than never smokers, while urinary cadmium remained elevated after smoking cessation, reflecting cumulative exposure. Plaque calcium content showed the strongest positive association with GSM, whereas cadmium concentrations in blood, urine, and plaque tissue were not independently associated with continuous GSM. Higher fruit and vegetable intake was also positively associated with plaque echogenicity. Integrating cadmium biomarkers, plaque calcium content, smoking-related variables, and dietary factors substantially improved prediction of GSM ≥ 75, achieving excellent discrimination (AUC = 0.870) with 85.0% classification accuracy. These findings suggest that carotid plaque echogenicity reflects the combined influence of local tissue mineralization, environmental exposures, and lifestyle-related factors. This integrative approach may improve our understanding of carotid plaque biology and support future strategies for risk assessment and clinical stratification of patients with carotid atherosclerosis.

## 1. Introduction

Cardiovascular diseases (CVDs) remain the leading cause of mortality worldwide, accounting for nearly one-third of all deaths [1,2]. Carotid atherosclerosis is one of the most clinically relevant manifestations of systemic vascular disease and accounts for approximately 20–25% of ischemic strokes [3]. Atherosclerotic plaques are in dynamic communication with the circulating blood. Throughout plaque development, bioactive molecules may be released from the lesion into the bloodstream, providing insights into the biological processes underlying plaque formation, progression, stability, and the consequent risk of cardiovascular and cerebrovascular events [4,5]. Moreover, accumulating evidence indicates that both the presence and specific characteristics of carotid plaques reflect the burden of atherosclerotic disease in other vascular territories and may therefore be regarded as systemic markers of atherosclerosis [5]. Gray-Scale Median (GSM), derived from B-mode ultrasonography, is a noninvasive surrogate marker of plaque composition [6]. Low-GSM plaques are generally characterized by lipid-rich necrotic cores, inflammatory cell infiltration, and intraplaque hemorrhage, representing a vulnerable plaque phenotype associated with an increased risk of plaque rupture and subsequent cerebrovascular events. Embolic or ischemic events arising from vulnerable carotid plaques may create conditions in which cortical spreading depolarizations occur, particularly within peri-infarct tissue. Experimental studies have shown that cortical spreading depression induces epigenetic chromatin remodeling, including altered H3K4 and H3K9 methylation and reduced expression of the histone methyltransferases MLL and SET7, suggesting a role in post-ischemic transcriptional regulation [7]. Conversely, highly echogenic plaques are typically enriched in fibrous and calcified components and represent a more stable plaque phenotype, with a lower propensity for plaque rupture and a reduced risk of ischemic stroke [8,9,10]. Our previous studies demonstrated that low GSM is associated with symptomatic carotid artery disease, identified monocyte chemoattractant protein-1 (MCP-1) as a biomarker of plaque instability, and highlighted the potential role of the endothelin-1 axis and lipid metabolism in carotid plaque stability, providing the foundation for the present investigation of environmental determinants of plaque phenotype [11,12,13]. Nevertheless, the biological and environmental factors that determine plaque echogenicity remain incompletely understood [6]. Environmental toxicants have emerged as important contributors to cardiovascular disease [14,15,16]. Among them, cadmium is of particular concern because of its widespread distribution and exceptional persistence in the human body [17,18]. Endothelial cells are considered the primary targets of cadmium-induced vascular toxicity [19,20]. Experimental studies have demonstrated that cadmium can induce endothelial dysfunction and cell death, leading to impaired endothelial integrity and subsequent vascular injury [19,20]. Chronic exposure to this trace element, even at low levels, has been associated with the development of severe cardiovascular disorders, including atherosclerosis, stroke, hypertension, and impaired cardiac function, and may contribute to increased cardiovascular mortality [21,22,23,24]. Cadmium exposure occurs primarily through tobacco smoking and dietary intake, particularly via cereals, vegetables, and contaminated food products [22,25,26,27,28,29]. Fruit and vegetables represent a particularly interesting component of cadmium exposure because they may serve both as a dietary source of cadmium and as markers of healthier dietary patterns. Although these foods can contribute to cadmium intake depending on environmental contamination, higher consumption is also associated with increased intake of antioxidants, fiber, and other bioactive compounds that may favorably influence cardiovascular health. Evaluating dietary habits alongside cadmium biomarkers may therefore provide a more comprehensive understanding of the complex relationships between environmental exposure and carotid plaque phenotype. Because the biological half-life of cadmium exceeds two decades, chronic exposure leads to progressive tissue accumulation and sustained biological effects long after exposure is reduced [17,18,23]. Experimental and epidemiological evidence demonstrates that cadmium contributes to atherosclerosis through multiple mechanisms, including endothelial dysfunction, oxidative stress, mitochondrial injury, chronic low-grade inflammation, macrophage activation, and disturbances in extracellular matrix turnover [19,20,30,31,32,33,34]. Cadmium may also exert molecular toxicity by altering protein conformation and protein-DNA interactions, leading to disturbed chromatin organization and enhanced DNA damage. Experimental studies in non-mammalian models have demonstrated that cadmium exposure disrupts chromatin-associated protein-DNA interactions, increases chromatin accessibility, promotes DNA fragmentation, and induces cellular stress responses [35]. Cadmium exposure has been associated with increased carotid intima-media thickness, peripheral artery disease, coronary heart disease, ischemic stroke, and cardiovascular mortality [34,36,37,38,39,40,41]. Furthermore, experimental evidence suggests that cadmium may contribute to vascular calcification by disrupting intracellular calcium homeostasis [42] and increasing oxidative stress [28,42], both of which are recognized drivers of osteogenic phenotypic switching of vascular smooth muscle cells [43,44,45]. In addition, cadmium has been shown to modulate matrix metalloproteinase activity, collectively favoring vascular remodeling and mineralization [28,46]. Because cadmium exhibits complex toxicokinetics, different biological matrices provide complementary information on exposure. Blood cadmium primarily reflects recent or ongoing exposure, whereas urinary cadmium is widely regarded as a biomarker of long-term cumulative body burden. In contrast, cadmium measured directly within carotid plaques may reflect local tissue accumulation associated with plaque development. Simultaneous assessment of all three compartments therefore enables a more comprehensive evaluation of both systemic exposure and local cadmium deposition within atherosclerotic lesions.

Despite growing evidence implicating cadmium in cardiovascular pathology, studies that simultaneously evaluate multiple cadmium compartments and their relationships with carotid plaque characteristics remain exceptionally limited. In particular, investigations quantifying cadmium directly within surgically excised carotid plaques are scarce. Moreover, little is known about how cadmium burden interacts with smoking history, dietary factors, and tissue mineralization to shape ultrasound-derived plaque phenotypes. Given that cardiovascular diseases result from cumulative interactions between environmental exposures and biological susceptibility, an exposome-oriented approach may provide a more comprehensive understanding of plaque stability than isolated biomarkers. We therefore hypothesized that carotid plaque echogenicity reflects the integrated effects of environmental cadmium exposure, smoking behavior, dietary habits, and local tissue mineralization. The aim of this study was to characterize cadmium concentrations in blood, urine, and carotid plaque tissue, investigate their associations with carotid plaque echogenicity assessed by GSM, and evaluate whether an integrated model incorporating cadmium biomarkers, smoking history, dietary habits, and plaque calcium content improves the prediction of a stable, highly echogenic plaque phenotype.

## 2. Materials and Methods

### 2.1. Study Population

A total of 80 patients who underwent CEA at the Department of Vascular Surgery and Transplantation, Medical University of Bialystok, were included in this cross-sectional study. All patients had carotid atherosclerotic plaques causing 50–99% internal carotid artery stenosis, as determined by computed tomography angiography (CTA) according to the North American Symptomatic Carotid Endarterectomy Trial (NASCET) criteria [47]. Patients were considered eligible for CEA if they had symptomatic carotid stenosis ≥50% or asymptomatic carotid stenosis ≥70%, irrespective of sex, in accordance with the European Stroke Organisation (ESO) guidelines [48]. Patients were consecutively recruited between February 2024 and March 2025. All participants underwent overnight fasting before blood sampling. Blood and urine samples were collected within one hour before CEA. Exclusion criteria included intracerebral hemorrhage, intracranial tumors, intracranial aneurysms, severe hepatic dysfunction, active infection, sepsis, systemic inflammatory response, autoimmune diseases, active malignancy, acute kidney injury, occupational exposure to cadmium or other heavy metals and refusal to provide informed consent. Based on previously published studies, a GSM threshold of ≥75 was used to define a highly echogenic plaque phenotype, whereas plaques with GSM <75 were classified as hypoechogenic plaques [49,50]. A detailed description of the GSM measurement technique has been published previously [11].

Before surgery, all participants underwent a detailed clinical interview to collect information on demographic characteristics, medical history, comorbidities, current pharmacological treatment, smoking habits, and dietary intake. Smoking-related data included current smoking status, former smoking status, smoking duration (years), the number of cigarettes smoked per day, time since smoking cessation, and cumulative smoking exposure expressed as pack-years. Dietary habits were assessed using a structured questionnaire. Participants reported their usual daily consumption of meat, fish, dairy products, fruit, vegetables, cereal products, sweets, and coffee using a six-point scale ranging from 0 to 5 servings per day, allowing assessment of dietary habits and potential dietary sources of exposure. Specifically, dietary habits were assessed using a dietary questionnaire developed specifically for the purposes of the present study. The questionnaire was not externally validated. Fruit and vegetable intake was quantified based on the self-reported number of daily servings consumed, with one serving defined according to the World Health Organization recommendation as approximately 80 g of the respective food product. Energy intake and other potential dietary confounders were not assessed in the present study.

The study was conducted in accordance with the Declaration of Helsinki, and approved by the Ethics Committee of Medical University of Bialystok (protocol code APK.002.108.2024, date of approval 22 February 2024). Written informed consent was obtained from all participants.

### 2.2. Laboratory Analyses

#### 2.2.1. Determination of Cd and Ca Concentrations in Blood/Serum, Urine and Carotid Plaque Tissue

Plaque samples (approximately 0.2–0.3 g) and blood samples (approximately 0.5 mL) were subjected to wet digestion with a mixture of trace-purity-grade 65% HNO_3_ and 30% HCl (9:1, *v*/*v*) using a microwave digestion system (Speedwave^®^ four Microwave Digestion System, Berghof Products + Instruments GmbH, Eningen, Germany). The microwave program consisted of heating at 160 °C and 40 bar with 80% power for 10 min, followed by heating at 190 °C and 40 bar with 90% power for 15 min, and a final cooling stage at 50 °C for 20 min. After digestion, the samples were cooled to room temperature and quantitatively transferred to glass tubes using ultrapure water, and the final volume was adjusted to 10 mL. One reagent blank was processed with each digestion batch of 11 samples to assess potential contamination. The blank was prepared in the same manner as the blood and plaque samples, using identical volumes of reagents. Urine samples were diluted according to the expected concentration range, and 0.5% (*v*/*v*) HNO_3_ (Suprapur^®^, Merck, Darmstadt, Germany) and 0.1% Triton X-100 were added to reduce surface tension. Serum samples were diluted with a solution containing 0.1% KCl and 0.1% CsCl according to the expected calcium concentration ranges.

##### Cadmium Determination

Cadmium concentrations were determined by graphite furnace atomic absorption spectrometry (GF-AAS) using a ContrAA^®^ 700 atomic absorption spectrometer (Analytik Jena AG, Jena, Germany). The primary analytical line at 228.802 nm was used. The optimized furnace program consisted of drying at 110 °C, pyrolysis at 700 °C, and atomization at 1600 °C. Quantification was performed using an external calibration curve prepared from aqueous standards. For this purpose, a 1 μg Cd/L working solution was prepared in Suprapur-grade nitric acid. Calibration standards ranging from 0.12 to 1.0 μg/L were prepared automatically by the autosampler through dilution. The limits of detection (LOD) and quantification (LOQ) for Cd were 0.020 and 0.065 μg/L, respectively. When necessary, samples were diluted with 0.5% HNO_3_ to ensure that Cd concentrations were within the calibration range.

##### Calcium Determination

Calcium concentrations were determined by high-resolution continuum-source atomic absorption spectrometry (HR-CS AAS) with atomization in an acetylene-nitrous oxide flame at an analytical wavelength of 422.673 nm, using a ContrAA^®^ 700 atomic absorption spectrometer (Analytik Jena AG, Jena, Germany). Quantification was performed using an external calibration curve over the range of 0.5–3.0 mg/L. All calibration standards were prepared manually in an aqueous solution containing 0.1% potassium chloride and 0.1% cesium chloride (Merck KGaA, Darmstadt, Germany). Because of the high Ca concentrations, all samples were diluted as necessary to bring the measured concentrations within the calibration range. The LOD and LOQ for Ca were 0.018 and 0.056 mg/L, respectively.

##### Laboratory Quality Control

Each sample was analyzed in triplicate, and reagent blanks were included to confirm that no contamination was introduced during sample preparation. Analytical accuracy and precision were assessed using certified reference and control materials, including Whole Blood Control L-1 (lot 2193), Urine Control L-1 (lot 2513), and ERM-BB184 Bovine Muscle. Recoveries obtained for Cd and Ca ranged from 94% to 104%, and the coefficients of variation (CVs) ranged from 5.7% to 13%. Detailed quality-control data, including assigned and measured concentrations, recoveries, and CVs, are presented in Table 1. The concentrations of Cd and Ca in carotid plaque tissue were expressed per gram of wet tissue, whereas urinary concentrations were normalized to creatinine.

### 2.3. Statistical Analysis

Statistical analyses were performed using Python 3.10 (SciPy, pandas, statsmodels, and scikit-learn libraries). Statistical significance was defined as a two-sided *p*-value < 0.05. The distribution of continuous variables was assessed using the Shapiro–Wilk test. Non-parametric methods were applied to variables that did not satisfy the assumption of normality. Associations between continuous variables were evaluated using Spearman’s rank correlation coefficient (ρ), or Pearson’s correlation coefficient (r) when data exhibited normal distributions. Correlation strength was classified as weak (0.10–0.29), moderate (0.30–0.49), or strong (≥0.50). Differences between independent groups were assessed using the Mann–Whitney U test. Effect sizes were reported as Cliff’s delta for non-parametric analyses and Cohen’s d for parametric analyses. Univariable linear regression models were used to evaluate continuous relationships, with the unstandardized regression coefficient (β) reported. For binary outcome analyses, GSM was dichotomized at GSM ≥ 75, and multivariable logistic regression models were constructed. Model discrimination was quantified using the area under the receiver operating characteristic curve (AUC), interpreted as poor (<0.70), acceptable (0.70–0.79), excellent (0.80–0.89), or outstanding (≥0.90). Classification accuracy was calculated at the optimal probability threshold determined from receiver operating characteristic (ROC) analysis. Model fit was evaluated using McFadden’s pseudo-R^2^, with values between 0.20 and 0.40 indicating excellent fit. Feature selection was performed using the Fast Correlation-Based Filter (FCBF) algorithm before fitting multivariable models. Because of the moderate sample size and the exploratory nature of the study, *p*-values were reported without adjustment for multiple testing and should be interpreted cautiously.

To assess the robustness of model discrimination, internal validation of the multivariable logistic regression models was performed using Harrell’s bootstrap optimism-correction procedure (1000 resamples). For each model, the apparent AUC from the development sample was compared with the bootstrap-derived optimism (the average overestimation of AUC due to overfitting), yielding an optimism-corrected AUC as an internal estimate of expected performance on new data.

## 3. Results

### 3.1. Patient Characteristics

Patients were categorized as non (never) smokers, current smokers, and former smokers. Full demographic and clinical baseline values are reported in Table 2.

### 3.2. Cadmium Concentrations Across Groups

**Figure 1:**
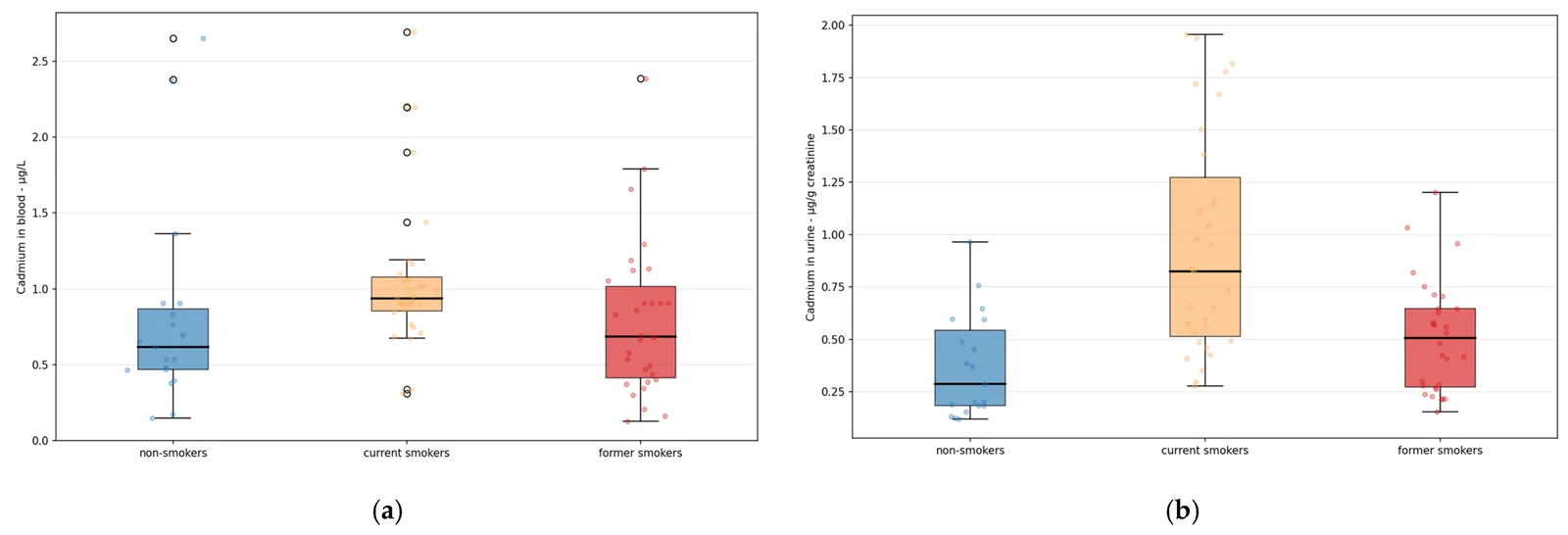
Blood (**a**) and urinary (**b**) cadmium concentrations according to smoking status. Data are presented for the study cohort of 80 patients with advanced carotid atherosclerosis undergoing carotid endarterectomy. Boxplots show the median, interquartile range (IQR), and whiskers extending to 1.5 × IQR after outlier removal.

Cadmium concentrations in blood and urine differed across smoking-status groups (Figure 1). Current smokers exhibited significantly higher Cd(blood) than never smokers (Figure 1a) (median 1.02 vs. 0.62 μg/L; Mann–Whitney U = 166, *p* = 0.0105, Cliff’s δ = −0.436, moderate effect size). In contrast, no significant difference was observed between former smokers and never smokers (median 0.69 vs. 0.62 μg/L; *p* = 0.845, Cliff’s δ = −0.035). Additionally, current smokers had higher Cd(blood) than former smokers (median 1.02 vs. 0.69 μg/L; *p* = 0.036, Cliff’s δ = 0.314).

Urinary cadmium differed significantly across the three patient groups (Figure 1b). Current smokers exhibited markedly higher Cd(urine) than never smokers (median 0.837 vs. 0.287 μg/g creatinine; Mann–Whitney U = 104, *p* = 0.0001, Cliff’s δ = −0.647, large effect size). Former smokers also showed higher Cd(urine) than never smokers (median 0.506 vs. 0.287 μg/g creatinine; Mann–Whitney U = 184, *p* = 0.039, Cliff’s δ = −0.354, moderate effect size). In addition, current smokers had significantly higher Cd(urine) than former smokers (median 0.837 vs. 0.506 μg/g creatinine; Mann–Whitney U = 677, *p* = 0.0023, Cliff’s δ = 0.456, moderate effect size).

**Figure 2:**
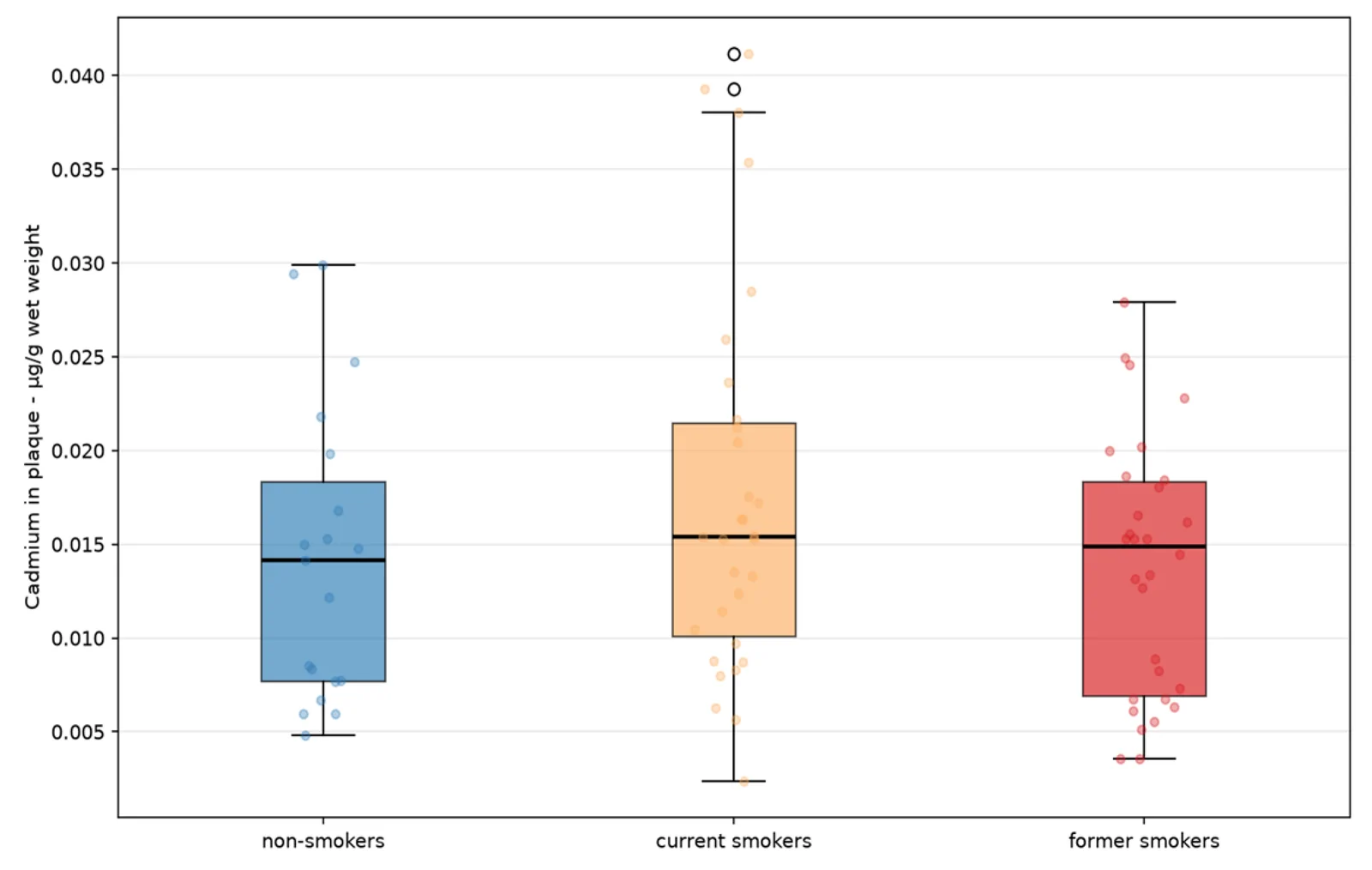
Cadmium concentrations in carotid plaque tissue according to smoking status. Data are presented for the study cohort of 80 patients with advanced carotid atherosclerosis undergoing carotid endarterectomy. Boxplots show the median, interquartile range (IQR), and whiskers extending to 1.5 × IQR after outlier removal.

Cadmium concentrations in carotid plaque tissue across different smoking cohorts are shown in Figure 2. Although pairwise comparisons did not reach strict statistical significance, distinct directional trends emerge that align with established toxicological principles. Current smokers exhibited the highest level of plaque cadmium accumulation, with a mean concentration of 0.0219 μg/g wet weight and the highest absolute tissue burden (max: 0.0967 μg/g). Conversely, non-smokers demonstrated the lowest baseline accumulation (mean: 0.0158 μg/g, median: 0.0142 μg/g). Interestingly, former smokers occupied an intermediate position (mean: 0.0171 μg/g, median: 0.015 μg/g).

### 3.3. Smoking Metrics and Cadmium

**Figure 3:**
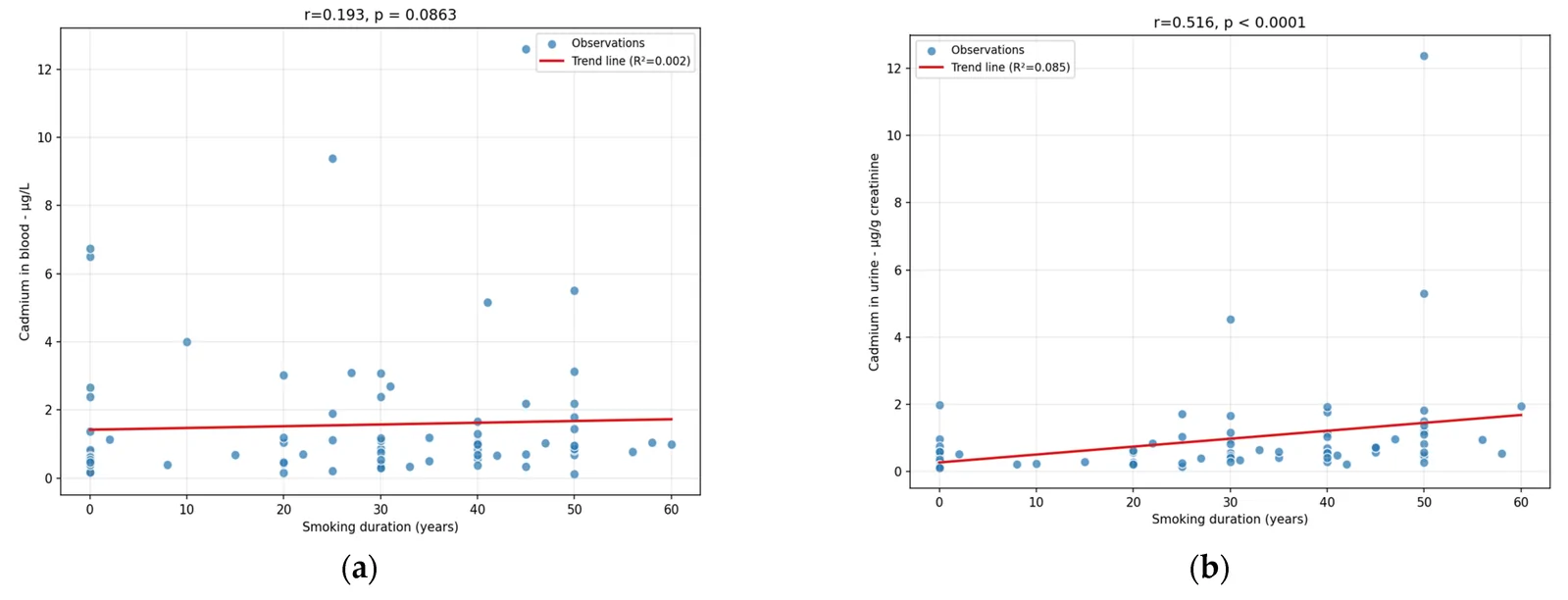

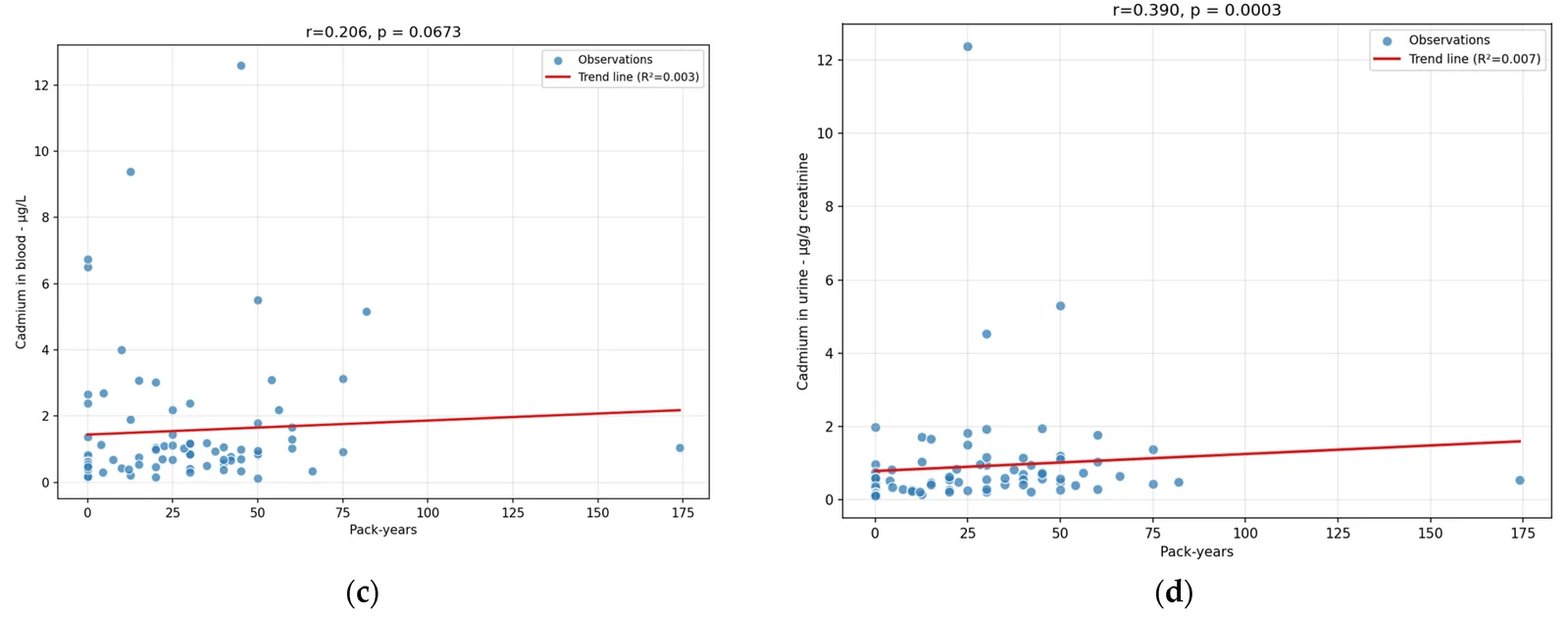
Associations between cumulative smoking exposure and cadmium concentrations in blood and urine. Data are presented for the study cohort of 80 patients with advanced carotid atherosclerosis undergoing carotid endarterectomy. (**a**) Smoking duration versus Cd(blood); (**b**) smoking duration versus Cd(urine); (**c**) pack-years versus Cd(blood); (**d**) pack-years versus Cd(urine).

Cadmium concentrations in urine and blood exhibited distinct relationships with smoking exposure metrics (Figure 3). Cd(urine) was positively and significantly correlated with both smoking duration (Spearman r = 0.516, *p* < 0.0001; Figure 3b) and pack-years (r = 0.390, *p* = 0.0003; Figure 3d). In contrast, Cd(blood) showed only weak positive associations with smoking duration (r = 0.193, *p* = 0.086; Figure 3a) and pack-years (r = 0.206, *p* = 0.067; Figure 3c), neither of which reached statistical significance.

**Figure 4:**
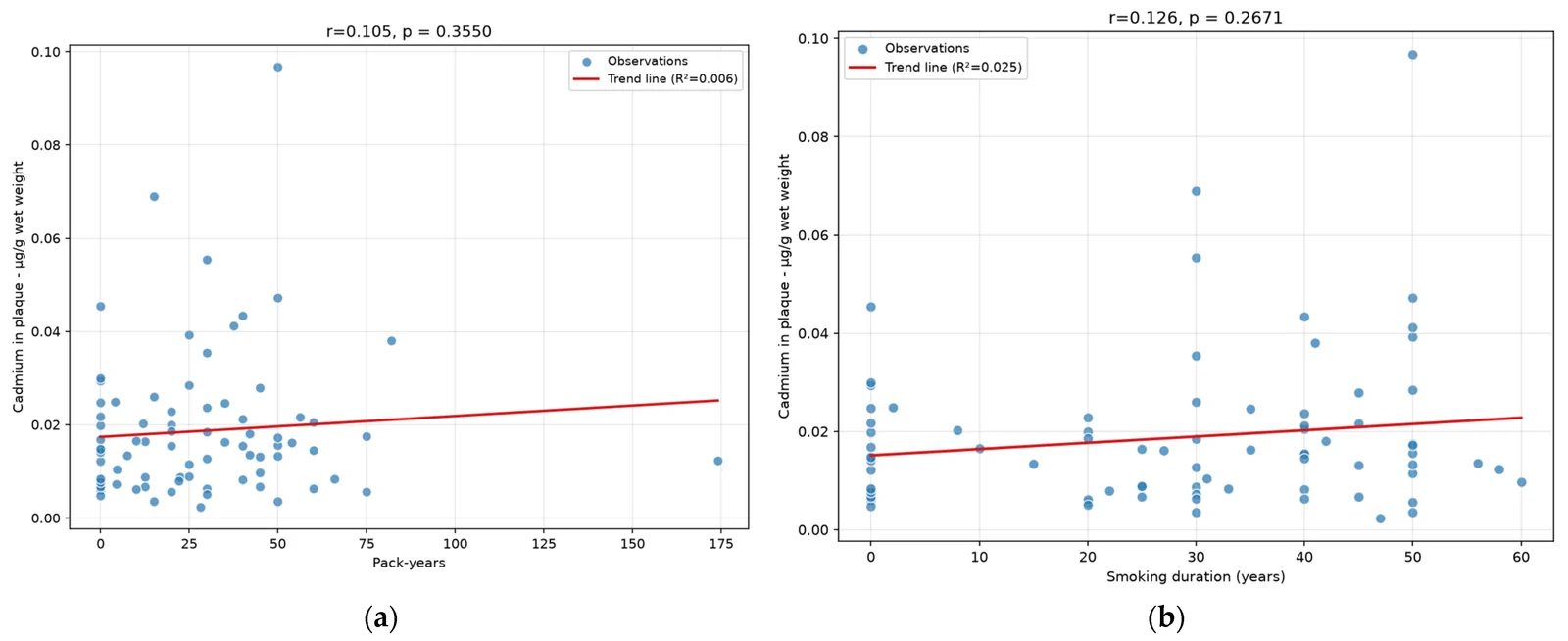
Associations between cumulative smoking exposure and carotid plaque cadmium concentrations. Data are presented for the study cohort of 80 patients with advanced carotid atherosclerosis undergoing carotid endarterectomy. (**a**) Pack-years versus Cd(plaque); (**b**) smoking duration versus Cd(plaque).

The relationship between cumulative smoking exposure and cadmium accumulation within the carotid plaque was evaluated using Spearman’s rank correlation analysis (Figure 4). For pack-years (Figure 4a), a positive directional trend was observed (ρ = 0.105, *p* = 0.355). Similarly, smoking duration (Figure 4b) displayed a parallel positive trend (ρ = 0.126, *p* = 0.267), where long-term smokers had an elevated mean tissue burden (0.0208 vs. 0.0169 μg/g wet weight).

### 3.4. Calcium Compartments and GSM

**Figure 5:**
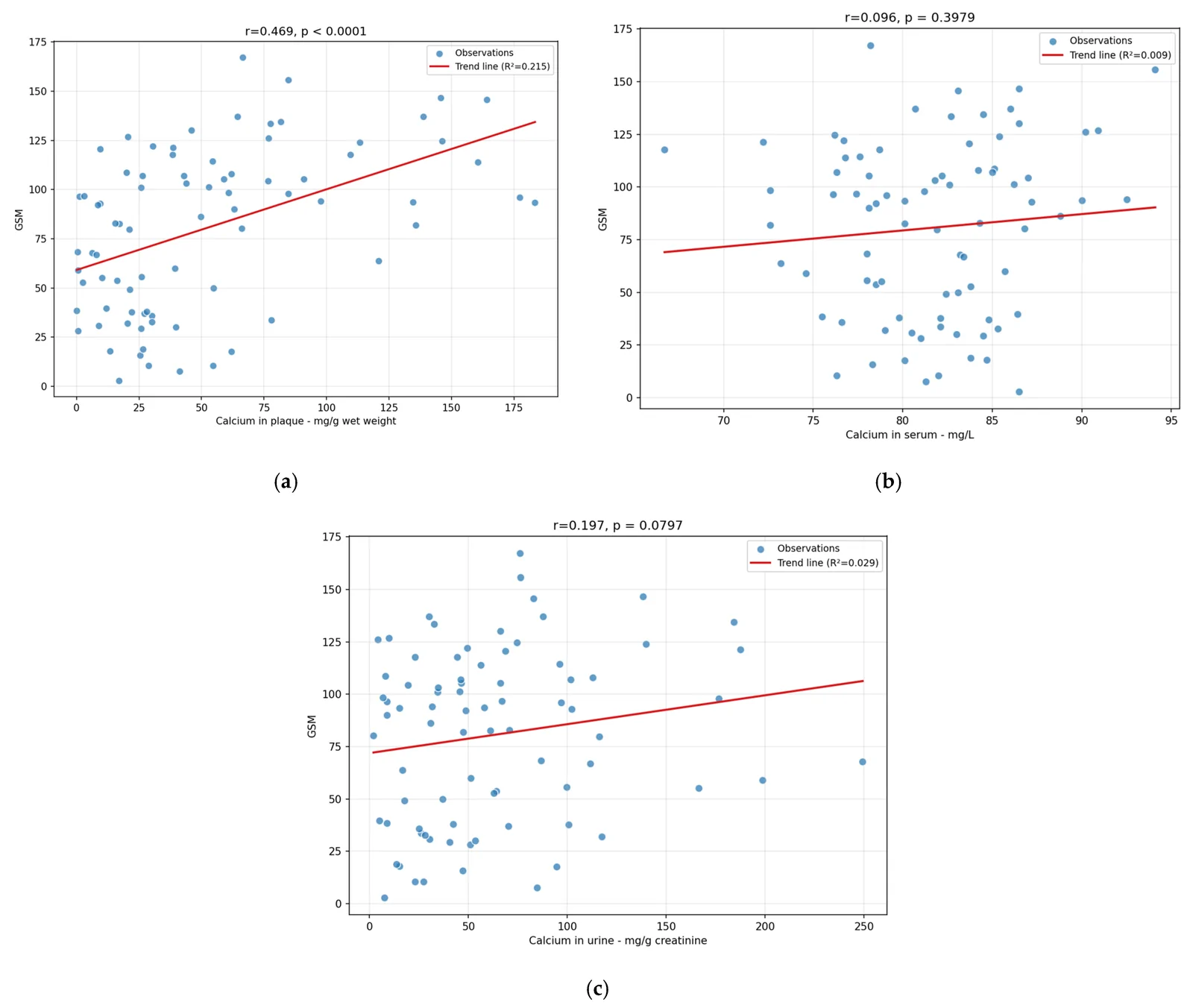
Associations between calcium concentrations in plaque tissue (**a**), serum (**b**), and urine (**c**) and carotid plaque echogenicity expressed as GSM. Data are presented for the study cohort of 80 patients with advanced carotid atherosclerosis undergoing carotid endarterectomy.

To determine whether carotid plaque echogenicity reflects localized mineral deposition or systemic calcium homeostasis, associations between continuous GSM and calcium concentrations were evaluated across three biological compartments: plaque tissue, blood serum, and urine (Figure 5).

Calcium in Plaque Tissue: Chemically quantified plaque calcium content showed the strongest association with ultrasound reflectivity (Figure 5a). Spearman rank correlation revealed a significant, moderate positive association (r = 0.469, *p* < 0.0001). In univariable linear regression, plaque calcium was a highly significant predictor, accounting for 21.5% of the total variance in GSM (R^2^ = 0.215, β = 0.410, *p* < 0.0001).

Calcium in Serum: In contrast to local tissue composition, systemic circulatory calcium levels showed no association with plaque echogenicity (Figure 5b).

Calcium in Urine: Urine calcium levels, measured via the urinary calcium-to-creatinine ratio, demonstrated a weak positive trend toward higher GSM that did not reach statistical significance (Figure 5c; r = 0.197, *p* = 0.080). Single-factor OLS regression (R^2^ = 0.029, β = 0.138, *p* = 0.131) did not show statistically significant results.

### 3.5. Dietary Variables and GSM

**Figure 6:**
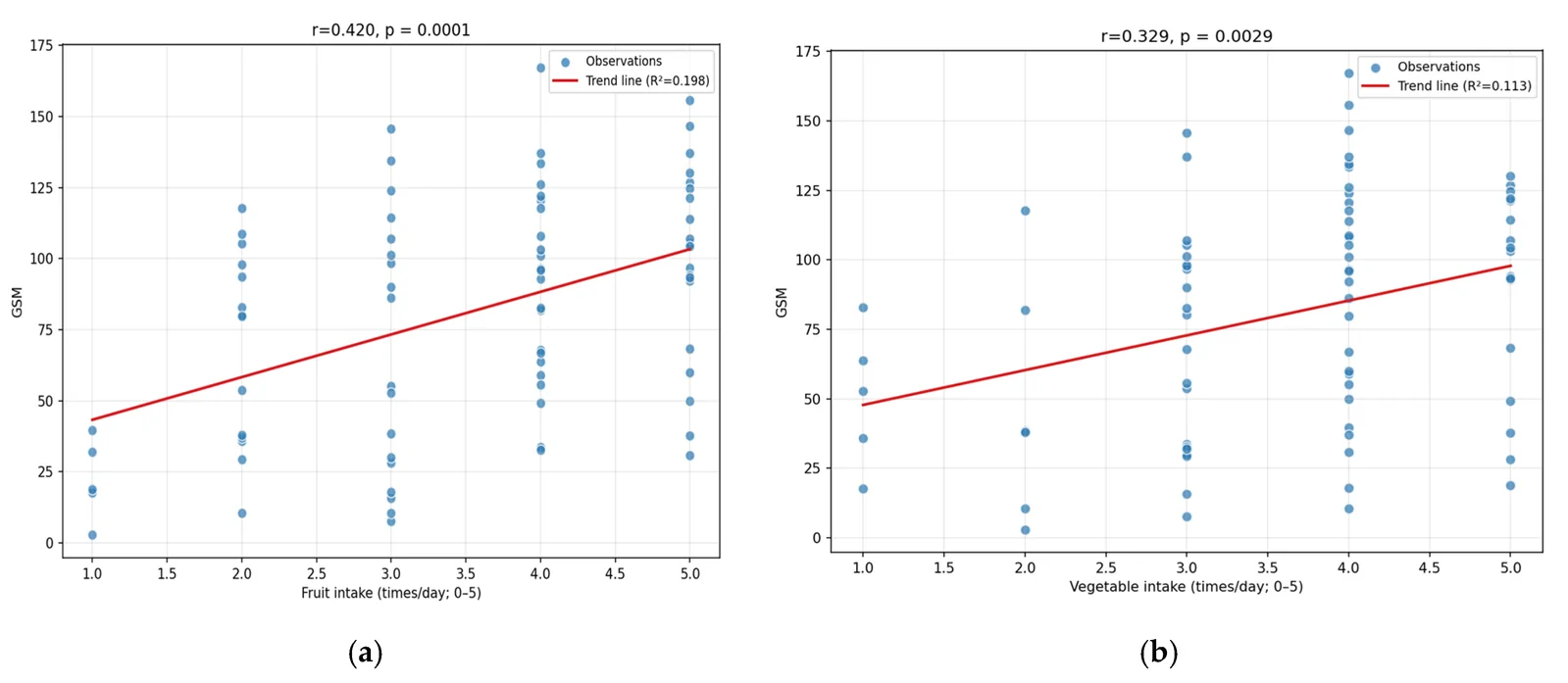
Associations between fruit (**a**) and vegetable (**b**) consumption frequencies and carotid plaque echogenicity. Data are presented for the study cohort of 80 patients with advanced carotid atherosclerosis undergoing carotid endarterectomy. Regression lines represent univariable linear models.

Among dietary frequency variables, fruit intake demonstrated the strongest positive correlation with GSM (Figure 6a; Spearman r = 0.420, *p* = 0.0001; linear regression R^2^ = 0.198, β = 15.0 GSM units per increment in fruit frequency; *p* < 0.0001). This was classified as a moderate positive correlation and was consistent across outlier-correction strategies applied to the dataset. Vegetable intake was also significantly positively associated with GSM, albeit more weakly (Figure 6b; r = 0.329, *p* = 0.003; R^2^ = 0.113, β = 12.5 GSM units per vegetable frequency increment, *p* = 0.002). Fish intake (r = 0.018, *p* = 0.877), dairy products (r = 0.170, *p* = 0.131), meat (r = −0.098, *p* = 0.388), sweets (r = 0.086, *p* = 0.447), and bread/cereals (r = −0.077, *p* = 0.500) showed no statistically significant correlation with GSM.

### 3.6. Cadmium Measures and GSM

**Figure 7:**
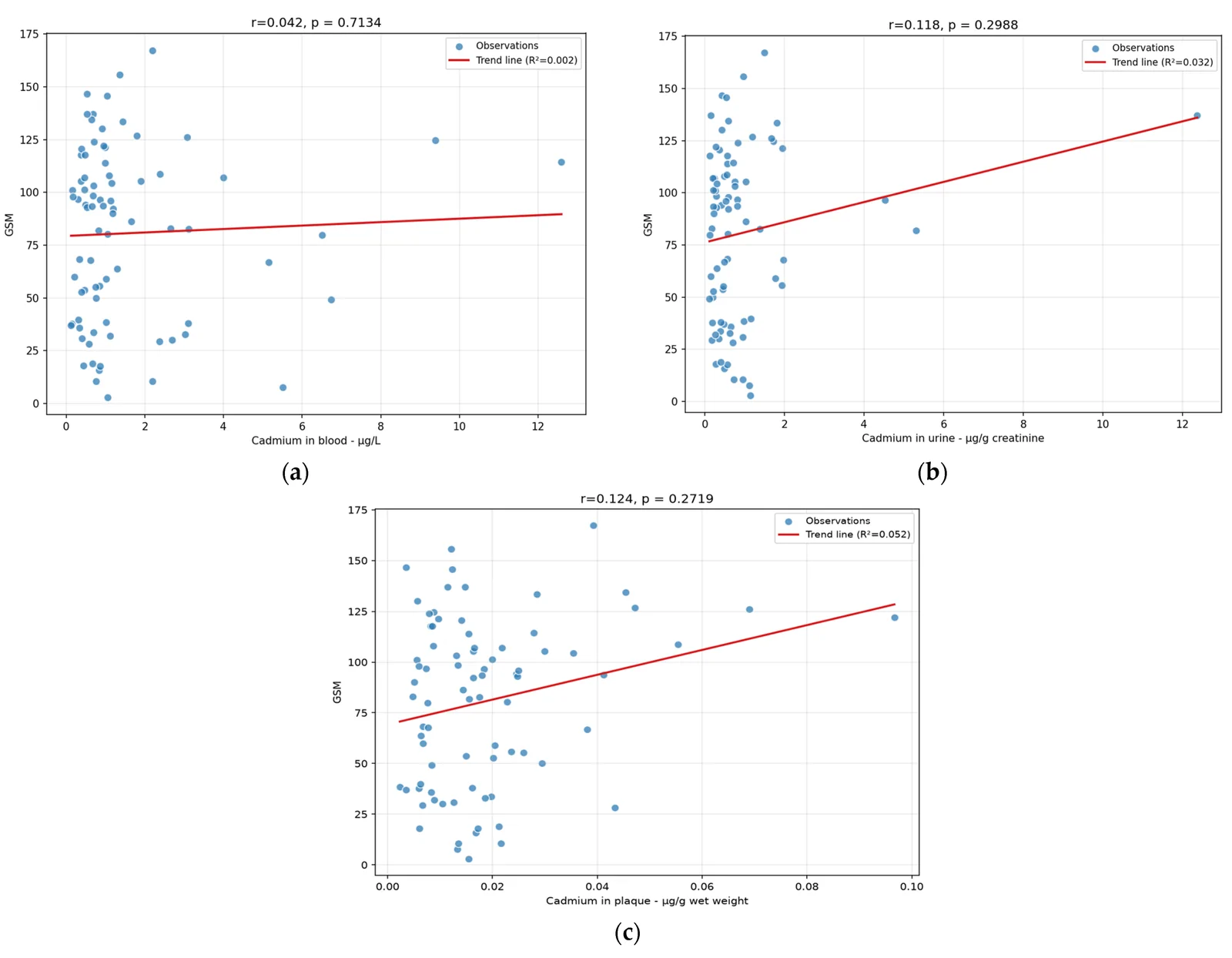
Associations between cadmium biomarkers and carotid plaque echogenicity. (**a**) Cd(blood); (**b**) Cd(urine); (**c**) Cd(plaque). Data are presented for the study cohort of 80 patients with advanced carotid atherosclerosis undergoing carotid endarterectomy.

To characterize relationships among the three cadmium compartments, we examined pairwise Spearman correlations between Cd(blood), Cd(urine), and Cd(plaque) (Table 3). Cd(blood) and Cd(plaque) were weakly but significantly correlated (ρ = 0.255, *p* = 0.023), whereas Cd(blood)-Cd(urine) (ρ = 0.156, *p* = 0.167) and Cd(urine)-Cd(plaque) (ρ = 0.188, *p* = 0.095) correlations did not reach statistical significance. Cadmium concentrations in all three measured compartments were not significantly correlated with continuous GSM (Figure 7). Specifically: Cd(blood) (Spearman r = 0.042, *p* = 0.713) (Figure 7a); Cd(urine) (r = 0.118, *p* = 0.299) (Figure 7b); Cd(plaque) (r = 0.124, *p* = 0.272) (Figure 7c). Linear regression confirmed the absence of meaningful linear associations (all R^2^ < 0.06; all regression *p* > 0.04). The within-subject variability of Cd(urine) [Cd(urine) SD] was similarly non-significant (r = 0.007, *p* = 0.950), as was the variability of Cd(blood) (r = 0.014, *p* = 0.901).

### 3.7. Multivariable Logistic Regression for GSM

Before presenting multivariable models, we evaluated single-feature logistic models for GSM ≥ 75 (excluding GSM itself as a predictor). Among individual features, calcium in plaque had the highest discriminative ability (AUC = 0.778, McFadden pseudo-R^2^ = 0.196, *p* = 0.0005), making it the strongest standalone marker. The best non-mineral single predictors were fruit intake (AUC = 0.658, *p* = 0.0108) and vegetable intake (AUC = 0.653, *p* = 0.0127), whereas single smoking and cadmium markers showed weaker discrimination (typically AUC about 0.50–0.59).

**Figure 8:**
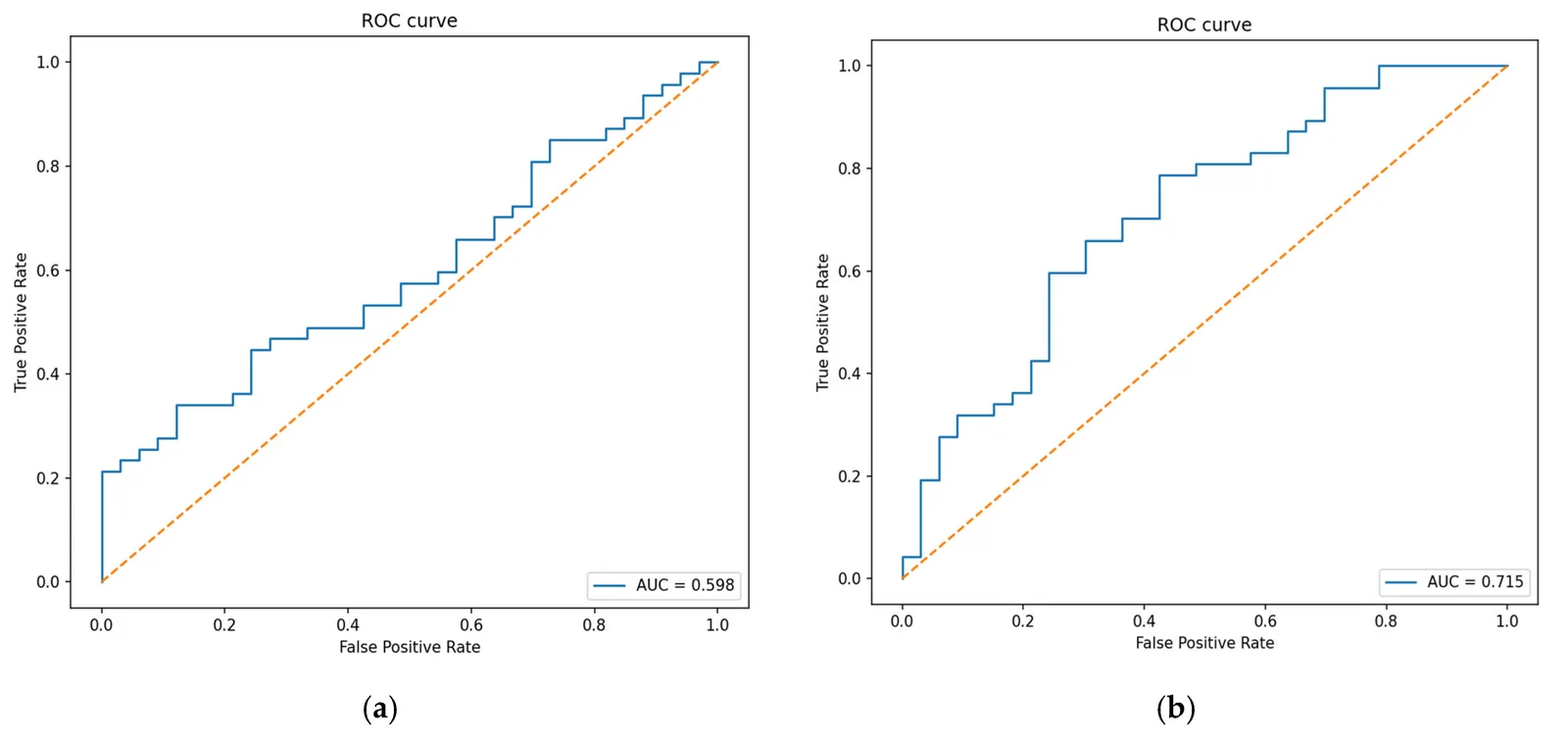
Receiver operating characteristic (ROC) curves for representative multivariable logistic regression models predicting highly echogenic carotid plaques. (**a**) Cadmium biomarkers only; (**b**) dietary and smoking variables. ROC analysis was performed in the study cohort of 80 patients with advanced carotid atherosclerosis undergoing carotid endarterectomy.

Table 4 summarizes the performance of the prespecified multivariable logistic regression models for predicting the binary outcome GSM ≥ 75. Among models based exclusively on cadmium biomarkers, the three-cadmium-marker model (blood, urine, and plaque cadmium) demonstrated limited discriminative ability (AUC = 0.598, McFadden pseudo-R^2^ = 0.052, Figure 8a). The addition of calcium content in plaque substantially improved model performance, increasing the AUC to 0.797 and the pseudo-R^2^ to 0.240. Smoking-related variables alone showed poor discrimination (AUC = 0.587, pseudo-R^2^ = 0.015), while dietary variables alone achieved moderate performance (AUC = 0.698, pseudo-R^2^ = 0.101). Combining dietary and smoking variables provided only a modest improvement over diet alone (AUC = 0.715, pseudo-R^2^ = 0.119, Figure 8b). Integration of smoking variables with cadmium biomarkers increased predictive performance relative to either domain alone (AUC = 0.682, pseudo-R^2^ = 0.095), indicating complementary information between exposure biomarkers and smoking history. The strongest parsimonious model incorporated calcium plaque content together with the seven dietary variables, yielding an AUC of 0.840 and a McFadden pseudo-R^2^ of 0.301.

**Figure 9:**
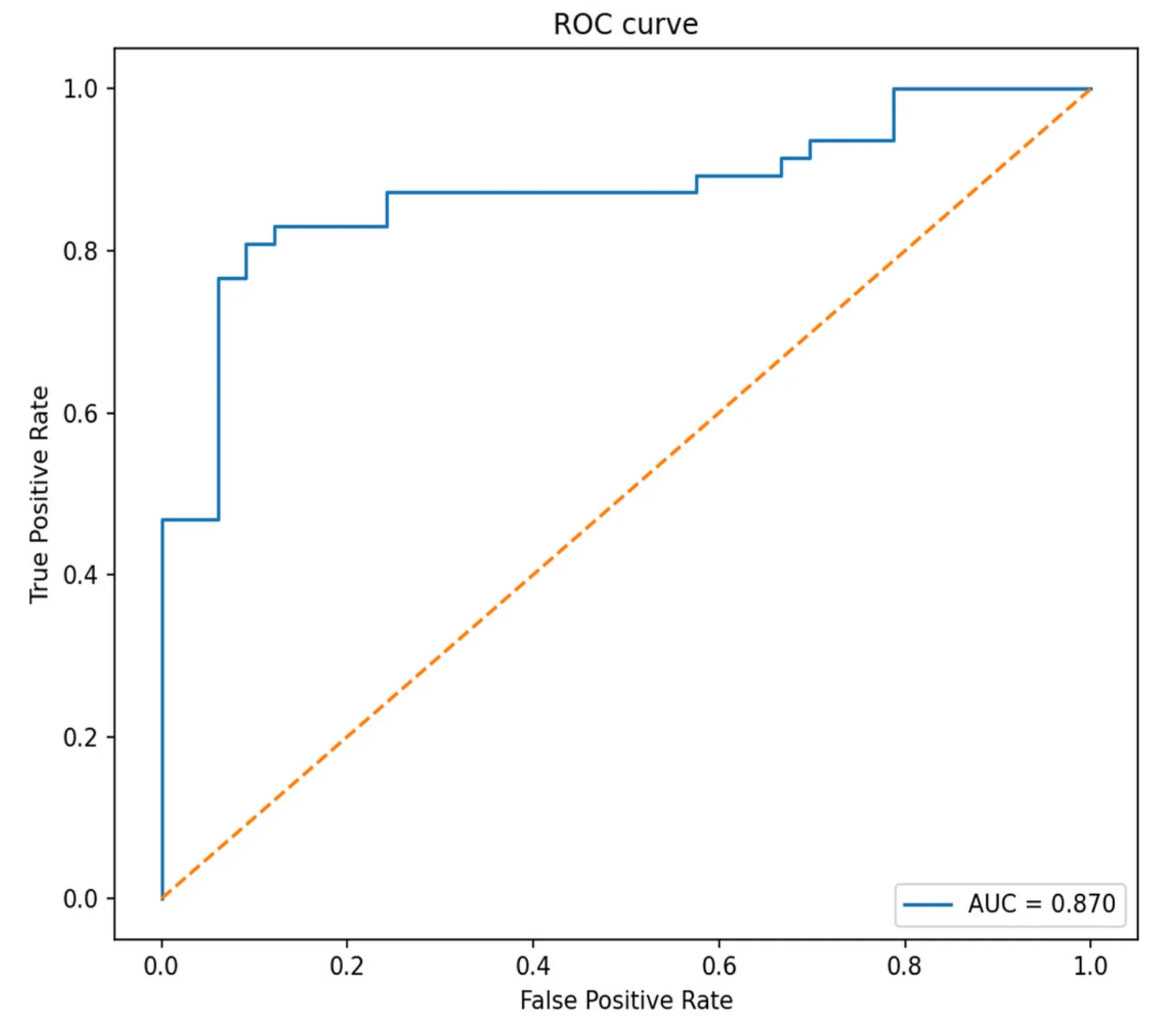
Receiver operating characteristic (ROC) curve of the final multivariable logistic regression model integrating plaque calcium, cadmium biomarkers, dietary variables, and smoking-related factors for predicting highly echogenic carotid plaques (GSM ≥ 75). ROC analysis was performed in the study cohort of 80 patients with advanced carotid atherosclerosis undergoing carotid endarterectomy.

The two top-performing configurations in the study emerged from the full integration of clinical, environmental, and behavioral features, presenting an interesting trade-off depending on the study’s design priorities. The maximum threshold-independent discrimination was achieved by the target full model presented in Figure 9 (17 predictors), which included all cadmium biomarkers, plaque calcium, diet, and smoking variables (AUC = 0.870, McFadden pseudo-R^2^ = 0.376, classification accuracy = 85.0%).

Conversely, expanding this framework to encompass all three calcium compartments (the 19-predictor model including blood and urinary calcium) yielded a fractionally lower discriminative value (AUC = 0.869, McFadden pseudo-R^2^ = 0.376) but achieved the highest peak classification accuracy across the entire study (86.2%). The choice between these two high-performing configurations ultimately reflects a design choice: the 17-predictor full model optimizes pure discriminative capacity (AUC) with fewer parameters, while the 19-predictor model maximizes point accuracy at the optimal classification threshold by capturing complete local and systemic calcium dynamics.

After internal validation using Harrell’s bootstrap optimism-correction, discrimination decreased for all models, confirming a degree of overfitting inherent in the developmental sample. Notably, the magnitude of this shrinkage was not simply proportional to the number of predictors: models anchored by plaque calcium. The strongest individual predictor retained comparatively stable performance after correction (cadmium biomarkers + plaque calcium: apparent AUC = 0.797, corrected AUC = 0.760; plaque calcium + dietary variables: apparent AUC = 0.840, corrected AUC = 0.774), whereas models combining multiple weaker, behaviorally correlated predictors in the absence of plaque calcium showed the largest optimism and the lowest corrected performance (diet + smoking: corrected AUC = 0.555; cadmium biomarkers + diet + smoking: corrected AUC = 0.563). The two fully integrated models retained apparent discrimination after correction (17-predictor model: corrected AUC = 0.741; 19-predictor model: corrected AUC = 0.719). This pattern suggests that plaque calcium acts as a stabilizing anchor predictor and that the incremental value of cadmium, smoking, and dietary variables is most robust when analyzed jointly with plaque calcium rather than in isolation. These findings were further corroborated by repeated k-fold cross-validation (5-fold CV repeated 100 times and 10-fold CV repeated 50 times), which yielded similar corrected AUC estimates across model configurations, supporting the robustness of the bootstrap-based conclusions.

## 4. Discussion

### 4.1. Principal Findings and Study Significance

The present study provides a comprehensive assessment of the relationships between environmental cadmium exposure, plaque calcium content, lifestyle-related factors, and carotid plaque echogenicity in patients with CEA. To our knowledge, this is among the few studies simultaneously evaluating cadmium concentrations in blood, urine, and surgically excised carotid plaques together with direct biochemical quantification of plaque calcium and ultrasound-derived GSM. The clinical relevance of a GSM ≥75 lies in its ability to identify a relatively more echogenic plaque phenotype, which is generally considered more stable and characterized by greater fibrous and calcified content. Moreover, categorizing plaques using this cut-off facilitates risk stratification and enables direct comparison with published studies employing the same approach. In the present study, a GSM threshold of 75 was selected based on published evidence investigating carotid plaque echogenicity and plaque stability. Published studies have consistently reported mean GSM values within a narrow range around this value, including 75.6 reported by Guo et al. [49], 71.75 in patients with stable atherosclerotic disease reported by Urbak et al. [50], and 78.4 in a large cohort of patients with carotid plaques reported by Della-Morte et al. [51]. In addition, Li et al. identified a GSM value of 75 as the optimal cut-off for predicting restenosis after CEA [52]. Taken together, these observations supported the selection of a GSM threshold of 75 in the present study. Nevertheless, this threshold has not yet undergone formal external validation in an independent cohort. The findings indicate that carotid plaque echogenicity represents a multifactorial phenotype reflecting the combined presence of local tissue mineralization, lifestyle-related factors, and cumulative environmental exposures. Plaque calcium content emerged as the strongest determinant of plaque echogenicity, whereas cadmium biomarkers provided complementary predictive value only when integrated with biological and lifestyle-related variables. Collectively, these findings suggest that carotid plaque characteristics are associated with the cumulative influence of multiple environmental and biological factors rather than by a single exposure, supporting an exposome-oriented framework for understanding plaque biology [28,53].

### 4.2. Smoking-Related Differences in Cadmium Distribution Across Biological Compartments

The distinct distribution of cadmium across biological compartments reflects their different toxicokinetic characteristics. This pattern suggests that Cd(blood) concentrations decline relatively rapidly after smoking cessation and gradually approach the levels observed in individuals who have never smoked. The observed differences between blood and urinary cadmium support their established roles as biomarkers of recent and cumulative exposure, respectively. Unlike Cd(blood), Cd(urine) remained elevated after smoking cessation, indicating persistent renal cadmium accumulation and long-term body burden rather than recent exposure. Together, these findings highlight the complementary value of both biomarkers, with Cd(blood) reflecting relatively recent exposure and Cd(urine) providing a reliable measure of cumulative cadmium retention [28,54,55,56]. Consistent with the correlation analysis (Table 3), only weak correlations were observed between cadmium concentrations measured in blood, urine, and carotid plaque tissue. This likely reflects the distinct biological significance of these matrices. Blood cadmium primarily reflects recent or ongoing exposure, whereas urinary cadmium is generally regarded as a biomarker of long-term cumulative burden. In contrast, cadmium measured within carotid plaques reflects local tissue accumulation over the course of plaque development. Consequently, only modest correlations between these biological compartments would be expected, as each represents a different temporal and biological dimension of cadmium exposure.

Unlike Cd(blood), Cd(plaque) did not normalize following smoking cessation, suggesting that cadmium may persist within vascular tissue for prolonged periods. However, because these differences did not reach statistical significance, the observed pattern should be interpreted cautiously. In addition to cumulative environmental exposure, local biological processes, including endothelial dysfunction, chronic inflammation, extracellular matrix remodeling, macrophage activity, and plaque composition, are also likely to influence cadmium accumulation within the arterial wall [31,42,57,58].

These findings are consistent with numerous epidemiological studies identifying cigarette smoking as the principal non-occupational source of cadmium exposure [28,55,56]. Tobacco plants readily accumulate cadmium from contaminated soils, and pulmonary absorption during smoking is substantially more efficient than gastrointestinal absorption from food, resulting in considerably greater body burdens among smokers [56,59]. The persistence of elevated Cd(urine) concentrations in former smokers is also consistent with the exceptionally long biological half-life of cadmium, estimated at 10–30 years, which results in prolonged retention of cadmium, particularly in the kidneys [54,56,59]. Similarly, the tendency toward higher cadmium concentrations in carotid plaques among current smokers may reflect long-term tissue accumulation, although confirmation of this hypothesis will require studies involving larger patient populations. Similar observations have been reported in studies demonstrating cadmium accumulation within human atherosclerotic plaques and suggesting that vascular tissue may serve as a long-term reservoir of this metal [28,36,37,38,39,40].

### 4.3. Cadmium Biomarkers in Relation to Smoking Duration and Pack-Years

The positive correlations between Cd(urine) and both smoking duration and pack-years further support the use of Cd(urine) as a biomarker of long-term cumulative exposure. In contrast, Cd(blood) exhibited only weak associations with cumulative smoking indices, reflecting its greater sensitivity to relatively recent exposure [55,60]. Additionally, cadmium concentrations measured directly in carotid plaques were not significantly associated with blood or urinary cadmium. This finding suggests that cadmium accumulation within advanced atherosclerotic plaques may depend not only on systemic exposure but also on local vascular processes [28,61]. Experimental evidence suggests that cadmium promotes atherosclerosis by enhancing macrophage accumulation within the arterial wall and promoting polarization toward the pro-inflammatory M1 phenotype. This process is accompanied by activation of the JAK2/STAT3 signaling pathway and increased production of pro-inflammatory cytokines, particularly IL-6 and TNF-α, thereby amplifying vascular inflammation and plaque progression [62].

A particularly important observation is the absence of a significant relationship between cadmium concentrations and continuous GSM values. Although cadmium exposure has consistently been associated with increased carotid intima-media thickness, peripheral artery disease, coronary heart disease, ischemic stroke, and cardiovascular mortality [28,60,63], considerably less is known about its relationship with plaque echogenicity. Therefore, the lack of a direct association observed in the present study does not necessarily exclude a pathogenic role of cadmium in plaque biology. Rather, the present findings may be consistent with the possibility that cadmium is involved predominantly in earlier stages of atherosclerosis, whereas GSM reflects the final structural balance between lipid accumulation, fibrosis, calcification, and intraplaque hemorrhage [28,42,61].

Experimental studies provide several biologically plausible mechanisms through which cadmium may contribute to vascular pathology. Previous studies have reported significantly higher cadmium accumulation within the cores of carotid plaques obtained from symptomatic patients than from asymptomatic patients, suggesting that local cadmium deposition may contribute to plaque vulnerability [64]. Cadmium disrupts intracellular calcium homeostasis [28,42,43,46,61] thereby promoting vascular remodeling, osteogenic differentiation of vascular smooth muscle cells, and subsequent vascular calcification [44,45,65]. Nevertheless, direct experimental evidence demonstrating that cadmium itself induces osteogenic differentiation of vascular smooth muscle cells remains limited. Consequently, cadmium should currently be regarded as an upstream modulator of pathways promoting vascular calcification rather than as a direct inducer of vascular mineralization [42,44].

### 4.4. Local Plaque Calcium, Rather than Systemic Calcium, Is the Major Determinant of Plaque Echogenicity

Vascular calcification is an active process that begins within inflammatory regions of the atherosclerotic plaque. During plaque progression, dying cells release matrix vesicles that promote calcium phosphate deposition, while vascular smooth muscle cells acquire osteoblast-like properties and produce a mineralized extracellular matrix [66]. Over time, small calcium deposits develop into larger calcified areas, increasing tissue mineralization. These changes enhance ultrasound reflectivity and provide a biological explanation for the higher GSM values observed in plaques with greater calcium content [67]. These findings indicate that plaque echogenicity is determined primarily by local tissue composition rather than systemic calcium homeostasis. Direct biochemical quantification of calcium within atherosclerotic plaques therefore appears to provide a more accurate measure of plaque mineralization than circulating calcium biomarkers and more closely reflects the biological processes responsible for plaque stabilization [45,65].

The observed association between plaque calcium content and GSM is biologically plausible. During the atherosclerotic process loss of cellular defense mechanisms, including mitochondrial dysfunction, excessive mitochondrial fragmentation, mitochondrial oxidative stress, impaired autophagy and mitophagy, as well as endoplasmic reticulum (ER) stress, may all contribute to vascular calcification [68]. As calcification progresses, plaques become increasingly fibrocalcific, resulting in greater ultrasound reflectivity, higher GSM values, and generally lower susceptibility to rupture. Consequently, plaque mineralization is widely regarded as one of the hallmarks of a more stable plaque phenotype, although the clinical significance may depend on the pattern and distribution of calcification [69,70].

Our findings are also consistent with histopathological studies demonstrating that echogenic carotid plaques contain greater proportions of fibrous tissue and calcification, whereas echolucent plaques are characterized by larger lipid-rich necrotic cores, inflammatory cell infiltration, and intraplaque hemorrhage [71,72]. The strong correlation observed between chemically quantified plaque calcium and GSM therefore provides biochemical confirmation of the histological basis of plaque echogenicity and further supports GSM as a biologically meaningful, non-invasive marker of plaque stability.

In contrast, neither serum nor urinary calcium concentrations were associated with plaque echogenicity. Serum calcium is maintained within a narrow physiological range through tightly regulated endocrine mechanisms involving parathyroid hormone, vitamin D, and calcitonin, whereas urinary calcium primarily reflects whole-body calcium balance, renal handling of calcium, dietary intake, and hormonal regulation [73]. Consequently, systemic calcium biomarkers are unlikely to reflect local mineral deposition occurring within the arterial wall, explaining their limited value for characterizing plaque composition.

To our knowledge, only a limited number of studies have directly quantified calcium in surgically excised human carotid plaques and related these measurements to ultrasound-derived plaque echogenicity. By combining direct biochemical determination of plaque calcium with preoperative GSM assessment, the present study provides further evidence that local tissue mineralization is one of the principal determinants of carotid plaque echogenicity. These findings also suggest that direct plaque calcium quantification may serve as a valuable reference standard in future studies evaluating imaging biomarkers of plaque vulnerability and stability.

### 4.5. Dietary Habits and Carotid Plaque Echogenicity

An additional noteworthy finding was the positive association between fruit and vegetable intake and plaque echogenicity. These observations suggest that habitual consumption of plant-based foods may be associated with a more stable carotid plaque phenotype. Although the cross-sectional design precludes causal inference, several biological mechanisms may explain this relationship. Fruits and vegetables are major dietary sources of antioxidants, polyphenols, vitamins, dietary fiber, potassium, magnesium, and other bioactive phytochemicals that attenuate oxidative stress, reduce chronic vascular inflammation, improve endothelial function, and favorably modulate vascular remodeling [74,75]. Since oxidative stress and chronic inflammation are key drivers of plaque progression and instability, the observed association between plant-rich dietary patterns and higher GSM values may be consistent with the known biological effects of these dietary components, but should not be interpreted as evidence of a direct causal relationship.

Interestingly, fruits and vegetables also represent one of the principal dietary sources of cadmium in the general population, particularly in regions where agricultural soils contain elevated cadmium concentrations [59,76]. However, despite contributing to dietary cadmium exposure, their overall cardiovascular benefits are likely to outweigh the potential adverse effects associated with low-level cadmium intake. This may be explained by the abundance of antioxidant and anti-inflammatory compounds capable of counteracting oxidative stress and vascular injury. Consequently, dietary cadmium exposure should not be interpreted independently of overall dietary quality, because foods containing cadmium also provide numerous nutrients and phytochemicals with well-established cardioprotective properties. This apparent paradox highlights the importance of evaluating the cumulative biological effects of whole dietary patterns rather than individual dietary components or contaminants in isolation.

In contrast, no significant associations were observed between plaque echogenicity and the consumption of meat, fish, dairy products, cereal products, or sweets. These findings suggest that overall dietary patterns may exert a greater influence on plaque composition than individual food groups. Nevertheless, these observations should be interpreted cautiously because dietary intake was assessed using a study-specific food frequency questionnaire. Higher consumption of plant-based foods may represent a marker of an overall healthier lifestyle characterized by more favorable dietary habits, lower cardiometabolic risk, and reduced vascular inflammation. Consistent with this interpretation, fruit and vegetable intake was identified by the Fast Correlation-Based Filter (FCBF) algorithm as an informative, non-redundant predictor of GSM ≥ 75, and both variables improved the predictive performance of the multivariable logistic regression models.

### 4.6. Cadmium Biomarkers and Plaque Echogenicity

Although chronic cadmium exposure has consistently been associated with carotid atherosclerosis, peripheral artery disease, ischemic stroke, and cardiovascular mortality [28,58,60,63], relatively few studies have examined its relationship with carotid plaque composition assessed by ultrasound. Consequently, the absence of a direct association between cadmium concentration and GSM observed in the present study should not be interpreted as evidence against a pathogenic role of cadmium in atherosclerosis. Previous studies investigating heavy metals have primarily focused on the presence and burden of carotid atherosclerosis rather than plaque echogenicity. Large population-based studies demonstrated positive associations between blood lead concentrations and carotid plaque prevalence and plaque burden [77,78], while a prospective study reported that chronic arsenic exposure was associated with greater carotid intima-media thickness and plaque score [79]. Consistent with these findings, a recent systematic review and meta-analysis also supported associations between cadmium exposure and subclinical carotid atherosclerosis, although the available evidence remained heterogeneous [80]. A recent report has demonstrated that cadmium accumulates extensively within carotid plaques, with tissue concentrations substantially exceeding those observed in the circulation [40]. Furthermore, cadmium has been shown to preferentially accumulate in rupture-prone regions of symptomatic plaques, supporting a potential role for local cadmium deposition in plaque vulnerability [40]. In the present study, however, plaque cadmium was not independently associated with plaque echogenicity, indicating that although cadmium may contribute to plaque progression, it is unlikely to represent a direct determinant of ultrasound-derived plaque phenotype.

Therefore, cadmium may represent a potential upstream modulator of pathways involved in vascular calcification rather than a direct inducer of vascular mineralization [42,44,65], although this hypothesis requires confirmation in prospective mechanistic studies.

Although cadmium biomarkers alone demonstrated poor discriminative performance, integrating cadmium concentrations with smoking history, dietary variables, and plaque calcium substantially improved prediction of a highly echogenic plaque phenotype. These findings indicate that cadmium biomarkers provide complementary information when evaluated together with other environmental and biological determinants, rather than acting as an isolated predictor of plaque stability.

This interpretation is consistent with the contemporary concept of the exposome, in which environmental toxicants interact with endogenous biological processes and lifestyle-related factors throughout life to influence chronic disease development [53].

Similarly, the absence of significant associations between within-subject variability of blood or urinary cadmium concentrations and GSM suggests that short- and medium-term fluctuations in cadmium exposure exert little influence on plaque echogenicity. Because carotid atherosclerosis develops over decades, plaque composition is more likely to reflect cumulative lifetime exposure than transient changes in circulating cadmium biomarkers [60].

It should also be emphasized that the present analyses evaluated only linear and monotonic relationships. Consequently, more complex non-linear associations between cadmium burden and plaque echogenicity cannot be excluded. For example, biological effects of cadmium may become apparent only after exceeding a certain exposure threshold or may differ across exposure ranges. Such non-linear dose–response patterns could partly explain why cadmium biomarkers were not independently associated with continuous GSM despite improving the predictive performance of the multivariable model. Future studies involving larger cohorts and dedicated non-linear modeling approaches are warranted to further investigate these potential relationships. Nevertheless, the multivariable logistic regression model based on the clinically established GSM threshold provided a robust and clinically interpretable framework for evaluating the combined contribution of environmental, biological, and lifestyle-related factors.

### 4.7. Integrated Prediction Models Support an Exposome-Oriented Approach

Cadmium biomarkers, smoking-related variables, and dietary factors each showed only modest predictive ability when analyzed separately, indicating that individual exposure markers do not adequately capture the biological complexity of carotid plaque composition. This pattern reflects a statistical distinction rather than a contradiction: a biomarker can show no independent association with a continuous outcome while nonetheless contributing meaningful predictive value within a multivariable framework capturing joint or complementary effects. This observation is consistent with the multifactorial nature of atherosclerosis, in which plaque development and remodeling result from lifelong interactions among environmental exposures [53,69].

A progressive improvement in model discrimination was observed following the sequential incorporation of plaque calcium, cadmium biomarkers, smoking variables, and dietary factors. These findings suggest that each domain contributes complementary rather than redundant biological information. In particular, the improvement observed after combining smoking history with cadmium biomarkers indicates that behavioral exposure metrics and biological exposure markers reflect different aspects of cadmium toxicokinetics. Whereas smoking history estimates cumulative external exposure, cadmium concentrations in blood and urine reflect internal exposure and body burden, providing complementary information that should not be considered interchangeable [54,55,56].

Interestingly, the inclusion of systemic calcium measurements (serum and urine) produced only minimal improvements in predictive accuracy, whereas local plaque calcium substantially enhanced model performance. This finding supports previous evidence indicating that plaque echogenicity is determined primarily by local tissue composition rather than systemic calcium homeostasis [71,72].

Collectively, these findings suggest that carotid plaque stability cannot be adequately explained by isolated biomarkers but instead reflects the cumulative effects of multiple interacting environmental and biological determinants. From a biological perspective, this observation is consistent with the concept of the exposome, which emphasizes that chronic diseases arise from lifelong interactions between environmental exposures and endogenous biological processes [53]. Rather than acting independently, environmental toxicants, smoking, diet, and local tissue remodeling appear to contribute jointly to plaque phenotype.

Importantly, our study extends this concept by demonstrating that the simultaneous integration of environmental biomarkers, lifestyle-related variables, and direct biochemical measures of plaque composition substantially improves prediction of plaque echogenicity compared with individual biomarkers alone. To our knowledge, this is the first study to combine cadmium concentrations measured simultaneously in blood, urine, and carotid plaque tissue with direct plaque calcium quantification within an exposome-oriented predictive framework. These findings support the growing view that environmental toxicology should move beyond single-exposure analyses toward multidimensional models integrating environmental, biological, and clinical determinants of cardiovascular disease [81].

### 4.8. Strengths and Limitations

The present study has several limitations. The cross-sectional design precludes causal inference. Accordingly, the observed associations between cadmium exposure, dietary factors, plaque calcium content, and plaque echogenicity should not be interpreted as evidence of causal relationships. The relatively small sample size limits statistical power, particularly for subgroup analyses involving plaque cadmium concentrations. Dietary assessment was based on food-frequency reporting rather than rigorous quantitative nutrient intake, and residual confounding cannot be excluded. Furthermore, biomarkers directly reflecting oxidative stress, inflammation, osteogenic signaling, or matrix remodeling were not measured, precluding mechanistic verification of the proposed biological pathways. Future prospective longitudinal and mechanistic studies are required to determine whether the associations observed in the present study reflect causal biological relationships or shared environmental and lifestyle determinants.

Despite these limitations, the study possesses several important strengths. It is among the first investigations simultaneously quantifying cadmium in blood, urine, and carotid plaque tissue together with biochemical determination of plaque calcium in the same patients. Furthermore, the integration of environmental exposure biomarkers, smoking metrics, dietary habits, tissue mineralization, and ultrasound-derived plaque characteristics represents an exposome-oriented approach that extends previous investigations based on single biomarkers. These findings provide new insight into the complex interactions between environmental toxicants and plaque biology and may contribute to improved risk stratification in patients with carotid atherosclerosis.

Although internal validation using bootstrap optimism-correction (Harrell’s method, 1000 resamples) confirmed that model discrimination decreased after adjustment for overfitting, the magnitude of this shrinkage varied considerably across model configurations (Table 4). Models lacking plaque calcium and combining several weaker, partially correlated exposure and behavioral predictors (e.g., diet + smoking, cadmium + diet + smoking) showed the largest optimism and the lowest corrected AUC (≈0.55–0.56), indicating limited robustness and a substantial risk of overfitting in the present sample size. In contrast, models incorporating plaque calcium retained comparatively strong corrected discrimination (corrected AUC 0.72–0.77 for the calcium-containing models, including both fully integrated 17- and 19-predictor configurations), suggesting these findings are less driven by chance. Nonetheless, no external validation in an independent cohort was performed, and the reported apparent AUCs, particularly for models without plaque calcium as an anchor predictor, should be regarded as optimistic estimates of true predictive performance. Larger, prospective, externally validated cohorts are warranted to confirm which combinations of predictors provide genuine, reproducible incremental value.

## 5. Conclusions

This study provides a comprehensive evaluation of the relationships between environmental cadmium exposure, plaque calcium content, lifestyle factors, and carotid plaque echogenicity in patients undergoing carotid endarterectomy. Cadmium concentrations measured in blood, urine, and carotid plaque were not independently associated with plaque echogenicity assessed by GSM. It should be emphasized that this lack of independent association reflects the absence of a direct, isolated statistical relationship between individual cadmium biomarkers and continuous GSM and is conceptually distinct from the substantial improvement in discriminative performance observed when cadmium biomarkers were incorporated into multivariable models. These two findings are not contradictory: a biomarker may lack independent explanatory value in isolation while still contributing complementary, non-redundant information that enhances overall model performance. In contrast, plaque calcium content emerged as the strongest determinant of a highly echogenic plaque phenotype, highlighting the central role of local mineralization in shaping ultrasound plaque characteristics. Simultaneous assessment of cadmium in three biological compartments together with plaque calcium quantification represents a novel aspect of the present study. This multidimensional, exposome-oriented approach may improve future studies investigating environmental determinants of atherosclerotic plaque stability. Prospective longitudinal studies involving larger populations and incorporating biomarkers of oxidative stress, inflammation, osteogenic signaling, and extracellular matrix remodeling are warranted to further investigate the potential interactions between chronic cadmium exposure, vascular calcification, and plaque vulnerability. These findings highlight the potential value of incorporating environmental biomarkers into multivariable, exposome-oriented approaches to cardiovascular risk assessment.

## Figures and Tables

**Table 1:** Analytical accuracy and precision of cadmium (Cd) and calcium (Ca) determination using certified reference materials. The analyses were performed in 80 patients with advanced carotid atherosclerosis treated with carotid endarterectomy.

Certified Reference Material	Reference Values	Measured Values ^1^	Recovery	Precision (CV) ^2^
Analytical Quality of Cd Measurements				
Whole Blood Control lyophilised L-1 LOT 2193 (RECIPE Chemicals + Instruments GmbH, München, Germany)	1.09–1.82 μg/L (mean 1.46 μg/L)	1.38 ± 0.10 μg/L	94%	7.2%
Urine Control lyophilised L-1 LOT 2513 (RECIPE Chemicals + Instruments GmbH, München, Germany)	1.76–2.64 μg/L (mean 2.20 μg/L)	2.13 ± 0.12 μg/L	96%	5.7%
Standard Reference Material Bovine Muscle (ERM-BB184; European Commission—Joint Research Centre IRMM, Geel, Belgium)	2.2 ± 0.4 ng/g	2.3 ± 0.3 ng/g	104%	13.0%
Analytical Quality of Ca Measurements				
Trace Elements Serum L-1 LOT 1309438 Seronorm, Billingstad, Norway)	69–104 mg/L (mean 86 mg/L)	84 ± 5 mg/L	98%	5.9%
Urine Control lyophilised L-1 LOT 2513 (RECIPE Chemicals + Instruments GmbH, München, Germany)	14.3–20.2 μg/L (mean 17.3 μg/L)	15.3 ± 1.2 μg/L	88%	7.8%
Standard Reference Material Bovine Muscle (ERM-BB184; European Commission—Joint Research Centre IRMM, Geel, Belgium)	0.155 g/kg	0.150 ± 0.012 g/kg	97%	8.0%

^1^ Data are represented as mean ± SD. ^2^ Precision of measurements is expressed as a coefficient of variation (CV).

**Table 2:** Baseline demographic, clinical, biochemical, and pharmacological characteristics of the study population according to smoking status ^1^. Data are presented for the study cohort of 80 patients with advanced carotid atherosclerosis undergoing carotid endarterectomy.

	Former Smoker (n = 30)	Non-Smokers (n = 19)	Current Smokers (n = 31)
Age, years	68.7 (5.22)	71.0 (7.04)	66.8 (7.26)
Male sex, %	93.3	73.6	64.5
Body Mass Index (BMI, kg/m^2^)	27.94 (4.36)	28.29 (3.87)	25.85 (3.81)
Symptomatic patient, %	33.3	63.15	67.74
Obesity, %	36.6	31.5	16.1
Total cholesterol (TC, mg/dL)	120.0 (32.25)	116.9 (28.07)	130.3 (40.27)
Triglyceride (TG, mg/dL)	137.0 (63.21)	133.9 (60.50)	160.5 (94.11)
Hypertension, %	86.6	100.0	93.5
Heart failure, %	16.6	0.0	16.1
Arrhythmia, %	20.0	5.2	16.1
Ischemic heart disease (IHD, %)	26.6	47.3	19.3
Myocardial infarction (MI, %)	6.6	21.0	9.6
Percutaneous coronary intervention (PCI, %)	16.6	31.5	6.4
Coronary artery bypass grafting (CABG, %)	3.3	15.7	0.0
Peripheral artery disease (PAD, %)	40.0	52.6	45.1
Diabetes mellitus (DM, %)	33.3	52.6	45.1
Chronic kidney disease (CKD, %)	3.3	5.2	3.2
Transient ischemic attack (TIA, %)	3.3	15.7	9.6
Stroke, %	30.0	42.1	58.0
Hyperuricemia, %	23.3	21.0	0.0
Statins, %	86.6	100.0	80.6
Ezetimibe, %	20.0	31.5	12.9
Fibrate, %	3.3	5.2	3.2
Angiotensin-converting enzyme inhibitors (ACEI, %)	60.0	73.6	64.5
Angiotensin receptor blockers (ARB, %)	16.6	21.0	16.1
β-Adrenergic receptor antagonist (B-blockers, %)	53.3	63.1	61.2
Calcium channel blockers (Ca-blockers, %)	43.3	47.3	51.6
Loop diuretics, %	10.0	10.5	12.9
Mineralocorticoid receptor antagonists (MRA, %)	16.6	15.7	12.9
Thiazide and thiazide-like diuretic, %	36.6	21.0	35.4
Metformin, %	30.0	42.1	22.5
Insulin, %	6.6	0.0	6.4
Flozins (SGLT2 inhibitors, %)	20.0	5.2	19.3
Aspirin (ASA, %)	66.6	89.4	83.8
Clopidogrel, %	13.3	26.3	16.1
Anticoagulant therapy, %	10.0	10.5	16.1
Nootropic drug, %	16.6	31.5	16.1

^1^ Clinical and biochemical characteristics of participants. Categorical data are presented as percentages (%), continuous variables are presented as means (SD) if distributed normally or as medians (IQR) for non-normally distributed data.

**Table 3:** Spearman correlation matrix between cadmium concentrations measured in blood, urine, and carotid plaque tissue. Correlation analyses were performed in the study cohort of 80 patients with advanced carotid atherosclerosis undergoing carotid endarterectomy.

	Blood	Urine	Plaque
Blood	X	0.156 (*p* = 0.1667)	0.255 (*p* = 0.0226)
Urine	0.156 (*p* = 0.1667)	X	0.188 (*p* = 0.0945)
Plaque	0.255 (*p* = 0.0226)	0.188 (*p* = 0.0945)	X

**Table 4:** Performance of multivariable logistic regression models for prediction of high plaque echogenicity (GSM ≥ 75). Model performance is reported as the ROC curve AUC, Optimism-corrected AUC obtained via Harrell’s bootstrap optimism-correction procedure (1000 resamples), calculated as apparent AUC minus the mean bootstrap-estimated optimism, the McFadden pseudo-R^2^, and classification accuracy at the optimal threshold determined from the ROC curve. ROC analysis was performed in the study cohort of 80 patients with advanced carotid atherosclerosis undergoing carotid endarterectomy.

Model Description	Predictors	Apparent AUC	Optimism-Corrected AUC	McFadden Pseudo-R^2^	Accuracy at Best Threshold
Cadmium biomarkers (blood, urine, plaque)	3	0.598	0.556	0.052	56.3%
Smoking variables	6	0.587	0.586	0.015	61.3%
Dietary variables	7	0.698	0.598	0.101	68.8%
Smoking + cadmium biomarkers	9	0.682	0.560	0.095	67.5%
Diet + smoking variables	13	0.715	0.555	0.119	70.0%
Cadmium biomarkers + plaque calcium	4	0.797	0.760	0.240	77.5%
Plaque calcium + dietary variables	8	0.840	0.774	0.301	78.8%
Cadmium biomarkers + diet + smoking variables	16	0.734	0.5633	0.177	73.8%
Cadmium and Calcium biomarkers + diet + smoking variables	19	0.869	0.719	0.376	86.2%
Full model (cadmium biomarkers + plaque calcium + diet + smoking variables)	17	0.870	0.741	0.376	85.0%

## Data Availability

The data presented in this study are available on request from the corresponding author due to privacy.

## Abbreviations

The following abbreviations are used in this manuscript:

GSM	Gray-Scale Median
AUC	Area Under the Curve
CVDs	Cardiovascular diseases
CEA	Carotid Endarterectomy
MCP-1	Monocyte Chemoattractant Protein-1
CTA	Computed Tomography Angiography
NASCET	North American Symptomatic Carotid Endarterectomy Trial
ESO	European Stroke Organisation
Cd	cadmium
Ca	calcium
HNO_3_	nitric acid
HCl	hydrochloric acid
KCl	potassium chloride
CsCl	cesium chloride
GF-AAS	graphite furnace atomic absorption spectrometry with electrothermal atomization
FAAS	flame atomic absorption spectrometry
CV	coefficient of variation
ROC	receiver operating characteristic
FCBF	Fast Correlation-Based Filter
BMI	body mass index
TC	total cholesterol
TG	triglycerides
IHD	ischemic heart disease
MI	myocardial infarction
PCI	percutaneous coronary intervention
CABG	coronary artery bypass grafting
PAD	peripheral artery disease
DM	diabetes mellitus
CKD	chronic kidney disease
TIA	transient ischemic attack
ACEI	angiotensin-converting enzyme inhibitor
ARB	angiotensin II receptor blocker
B-blockers	beta-blockers
Ca-blockers	calcium channel blockers
MRA	mineralocorticoid receptor antagonist
SGLT2	sodium-glucose cotransporter 2 inhibitor
ASA	acetylsalicylic acid
SD	standard deviation
IQR	interquartile range
Cd(blood)	blood cadmium concentration
Cd(urine)	urinary cadmium concentration
Cd(plaque)	cadmium concentration in carotid plaque tissue
OLS regression	ordinary least squares regression
M1	classically activated macrophage (M1) phenotype
JAK2	Janus kinase 2
STAT3	signal transducer and activator of transcription 3
IL-6	interleukin-6
TNF-α	tumor necrosis factor-alpha
Ca(plaque)	calcium concentration in carotid plaque tissue
